# A Population-Based Survey on the Prevalence of Postoperative Nausea and Vomiting in the Qassim Region

**DOI:** 10.7759/cureus.104143

**Published:** 2026-02-23

**Authors:** Mohammed M Almadhi, Mohammed M Aboelseoud, Ahmad K AlGharras, Abdulaziz A Almadud, Emad A Mohamed, Ibrahim M Alshehri, Radwa M Geddawy, Wajd K AlGharras

**Affiliations:** 1 Anesthesia, King Fahad Specialist Hospital, Buraydah, SAU; 2 Anesthesia, Medical College, Al-Imam Mohmmad Ibn Saud University, Riyadh, SAU; 3 Anesthesia, Sohag University, Sohag, EGY; 4 Anesthesia, College of Medicine, Qassim University, Buraydah, SAU

**Keywords:** and retching, anesthesia, postoperative nausea and vomiting, prevalence, rhodes index of nausea, risk factors, vomiting

## Abstract

Introduction

Despite advances in anesthetic techniques and the routine use of prophylactic antiemetics, postoperative nausea and vomiting (PONV) remains a prevalent and distressing complication within the first 24 hours after surgery. It is associated with reduced patient satisfaction, prolonged hospital stays, and increased healthcare costs. This study aimed to assess the prevalence and severity of PONV and to identify associated risk factors among postoperative patients in the Qassim region of Saudi Arabia.

Methods

A cross-sectional survey was conducted among patients who had undergone surgery under anesthesia in the Qassim Province of Saudi Arabia. Data were collected using a structured, self-administered questionnaire covering demographic and clinical variables, along with the validated Rhodes index of nausea, vomiting, and retching (RINVR) to quantify PONV severity.

Design

This was a cross-sectional observational study using an online self-administered questionnaire in postoperative patients.

Results

A total of 392 patients participated. PONV (any nausea, vomiting, or retching) affected 72.7% of patients overall, with 66.8% reporting nausea, 42.9% vomiting, and 42.3% retching. PONV was more frequent and severe in female patients than in male patients (76.8% vs. 62.9% incidence, p=0.007). The type of anesthesia also significantly influenced PONV occurrence (p=0.003), with patients undergoing local anesthesia experiencing much lower rates than those receiving general anesthesia. Age and weight did not show a significant association with PONV in this sample.

Conclusion

PONV is common among surgical patients in the Qassim region, particularly in women and those under general anesthesia. These findings underscore the importance of recognizing high-risk patients and implementing targeted prophylactic strategies to reduce PONV and enhance postoperative recovery in Qassim and beyond.

## Introduction

Postoperative nausea and vomiting (PONV) is classically defined as nausea and/or vomiting occurring within 24 hours after surgery. It is among the most common and unpleasant complications of anesthesia and surgery and is reported as the second most frequent postoperative issue after pain [1]. PONV reduces overall patient comfort and satisfaction, may prolong hospital recovery, and ultimately increases healthcare costs [2]. Multiple studies have identified a variety of risk factors for PONV, including patient-specific factors (such as younger age, female sex, non-smoker status, history of motion sickness, and prior PONV) and anesthesia-related factors (notably the use of volatile anesthetics, nitrous oxide, mask ventilation, and perioperative opioids) [3]. Surgical factors, including the type and duration of the procedure, as well as inadequate perioperative hydration, have also been linked to higher PONV risk [3]. PONV may prolong a patient’s hospital stay by approximately 30 minutes on average [4]. Despite advances in anesthetic agents and surgical techniques, the incidence of PONV has remained relatively unchanged over recent decades [5]. Currently, antiemetics are routinely administered preoperatively and postoperatively; yet PONV continues to be a pressing clinical issue that affects a substantial proportion of surgical patients [6].

In light of these challenges, we conducted a population-based study in the Qassim region of Saudi Arabia to determine the current prevalence of PONV and identify associated risk factors in surgical patients. The primary objective was to quantify the prevalence of PONV in the first 24 hours after surgery and examine factors associated with its occurrence. Secondary objectives included assessing symptom severity using a validated nausea, vomiting, and retching severity scale and evaluating the relationships between PONV and patient demographics (age, gender, weight), as well as the type of anesthesia (general, regional, local, or sedation).

## Materials and methods

Study design

This study was a cross-sectional, community-based survey conducted in the Qassim region of the Kingdom of Saudi Arabia. We utilized an online self-administered questionnaire to collect data from postoperative patients. The study was carried out over a four-month period.

Study population

Eligible participants were residents of Qassim who had undergone surgical procedures involving any form of anesthesia. Inclusion criteria were broad: we included male and female patients of any age who underwent surgery under general, regional (e.g., spinal or epidural), or local anesthesia, or sedation. There were no restrictions based on previous PONV history, smoking status, body mass index, comorbidities (e.g., diabetes), type of surgery (elective or emergency), surgical technique, or bowel preparation. Participants were required to understand and complete the questionnaire in Arabic or English. Patients were excluded if their surgery was an outpatient procedure with less than 12 hours of postoperative observation or if they had cognitive or language barriers preventing understanding of the survey.

Sample size and sampling

We initially estimated that a sample of approximately 323 participants would be needed to estimate a PONV prevalence of ~30% with a 95% confidence level and ±5% precision. To account for subgroup analyses and possible nonresponse, we aimed to recruit at least 500 participants. Participants were recruited through convenience sampling, including online distribution of the survey link via social media, community forums, and QR codes posted in clinics and hospitals across the region. Ultimately, 392 participants completed the survey.

Data collection

Data were collected using a structured electronic questionnaire (via Google Forms). The questionnaire was divided into sections capturing: (1) demographic information (age, gender, and weight), (2) clinical and surgical information (type of surgery and type of anesthesia received), and (3) PONV outcomes measured by the Rhodes index of nausea, vomiting, and retching (RINVR) [7]. The RINVR is a validated instrument consisting of eight items that assess nausea, vomiting, and retching frequency, amount, and distress, each rated from 0 (none) to 4 (severe) [7]. The scores are summed to yield a total RINVR score ranging from 0 to 32, with higher scores indicating more severe nausea and vomiting symptoms [7]. In this study, any total RINVR score >0 was considered indicative of PONV (i.e., any presence of nausea, vomiting, or retching) [7]. For descriptive purposes, we categorized RINVR severity as: no symptoms (score 0), mild (1-8), moderate (9-16), severe (17-24), and great severity (25-32), based on the instrument’s descriptors [7]. No personally identifiable data were collected; all responses were anonymous, and participation was voluntary. The survey introduction explained the study’s purpose, how the data would be used, and assured participants of confidentiality. Informed consent was obtained electronically from each participant before proceeding to the questionnaire. Participants were free to discontinue the survey at any time without penalty. All responses were automatically recorded and stored securely in a password-protected electronic database accessible only to the research team.

Variables and definitions

The primary outcome was the occurrence of PONV within 24 hours post-surgery, defined as any RINVR score >0 (i.e., any nausea, vomiting, or retching). The secondary outcome was PONV severity as measured by the total RINVR score (0-32). Key independent variables included patient demographics (age, gender, and weight), type of anesthesia (general, spinal/epidural, regional, local, or sedation), and type of surgery. Age and weight were analyzed as categorical variables (age groups: <18, 18-24, 25-34, 35-44, 45-54, 55-64, ≥65 years; weight groups: <50, 50-69, 70-79, 80-89, and ≥90 kg). We also recorded whether the patient had any previous surgery (yes/no) as a characteristic of the sample.

Data analysis

All analyses were performed using IBM SPSS Statistics version 28 (IBM Corp., Armonk, NY). Categorical variables are presented as frequencies and percentages, and continuous variables are summarized as means with standard deviations or medians with interquartile ranges, as appropriate to their distribution. Bivariate comparisons were conducted to explore factors associated with PONV. Chi-square tests (or Fisher’s exact test when expected cell counts were low) were used for categorical variables, and independent-samples t-tests or nonparametric equivalents (Mann-Whitney U test for two groups; Kruskal-Wallis test for multiple groups) were used for continuous or ordinal outcomes. Spearman’s rank correlation was used to assess the relationship between age and total RINVR score. Total RINVR scores are reported as medians with interquartile ranges. A p-value <0.05 was considered statistically significant.

Ethical considerations

The study protocol was reviewed and approved by the Research Ethics Committee of Qassim Health Cluster (ethical approval number 607-47-2236; committee registration number H-04-Q-001) prior to data collection. All participants provided informed consent at the beginning of the online survey. The consent form described the study’s purpose and procedures, emphasized that participation was voluntary, and stated that responses would be kept confidential and used solely for research purposes. No personal identifiers were collected. All data were stored in a secure, password-protected electronic system, with access limited to the research team.

## Results

**Figure 1:**
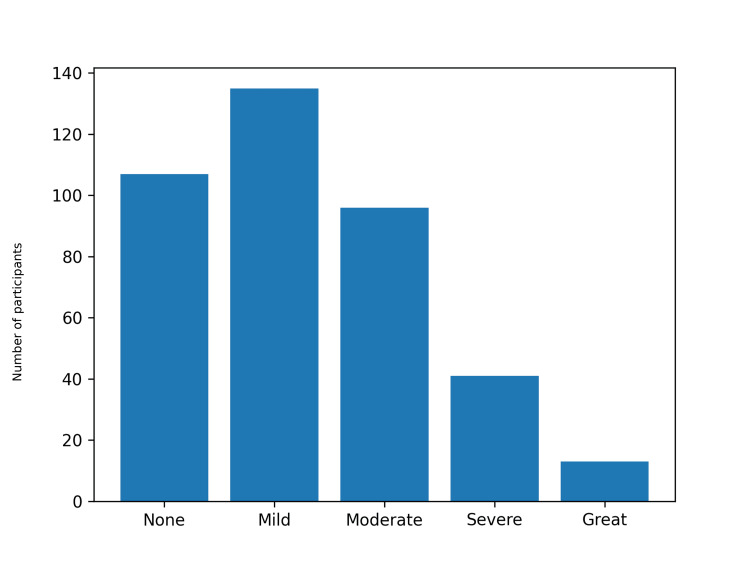
RINVR severity distribution Bars show the number of participants in each severity category (n=392) [7]. RINVR: Rhodes index of nausea, vomiting, and retching

**Figure 2:**
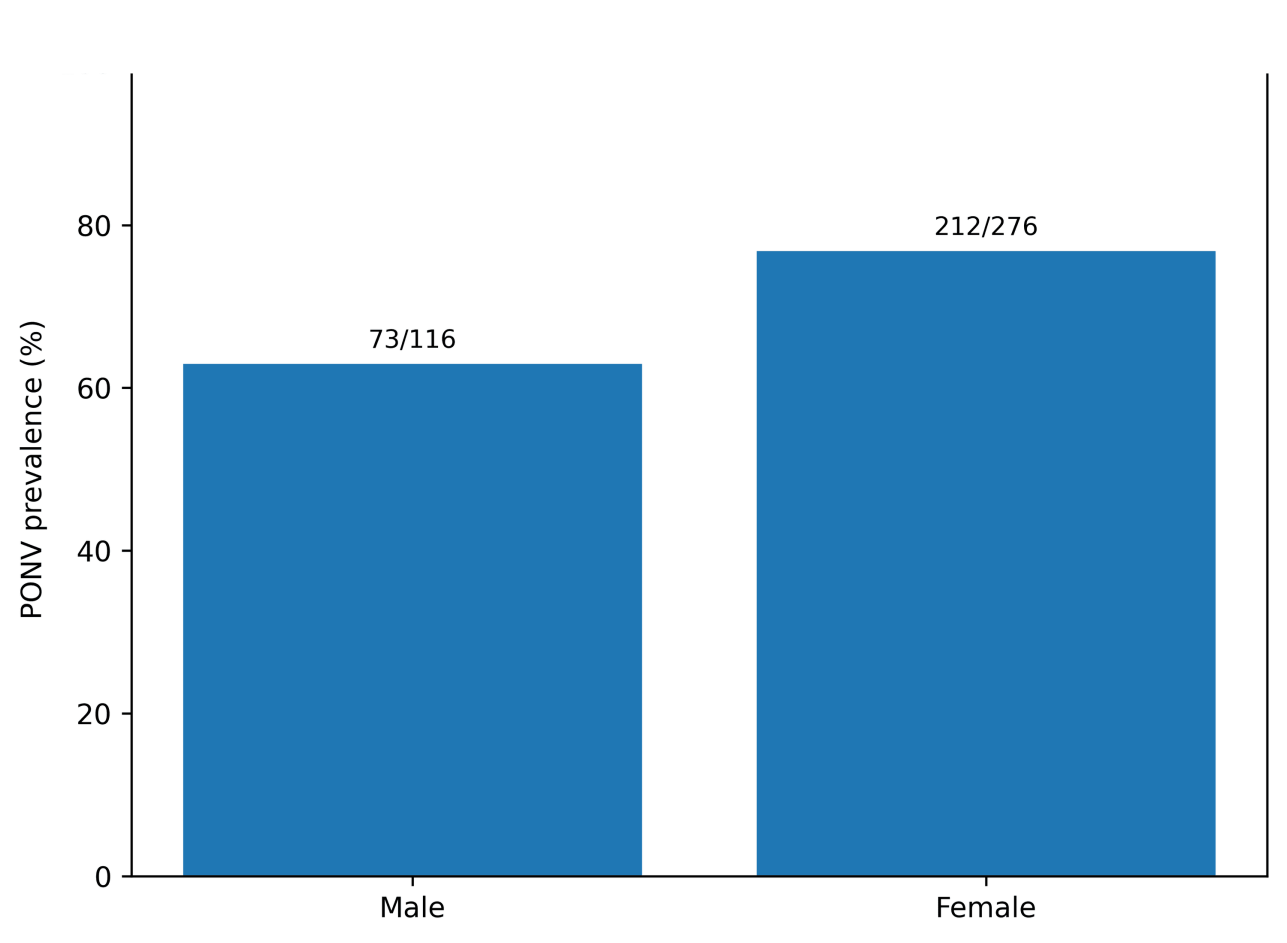
PONV prevalence by gender Bars show the percentage of participants with PONV (RINVR total >0) by gender (n=392) [7]. PONV: postoperative nausea and vomiting; RINVR: Rhodes index of nausea, vomiting, and retching

**Figure 3:**
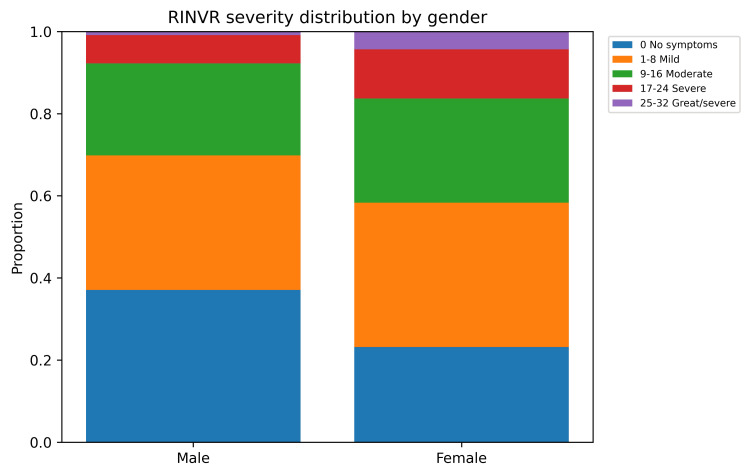
PONV severity distribution by gender Stacked bars show the proportion of male and female patients in each RINVR severity category (none, mild, moderate, severe, and great/severe). Female patients had a higher overall incidence of PONV and greater symptom severity compared with male patients [7]. PONV: postoperative nausea and vomiting; RINVR: Rhodes index of nausea, vomiting, and retching

**Figure 4:**
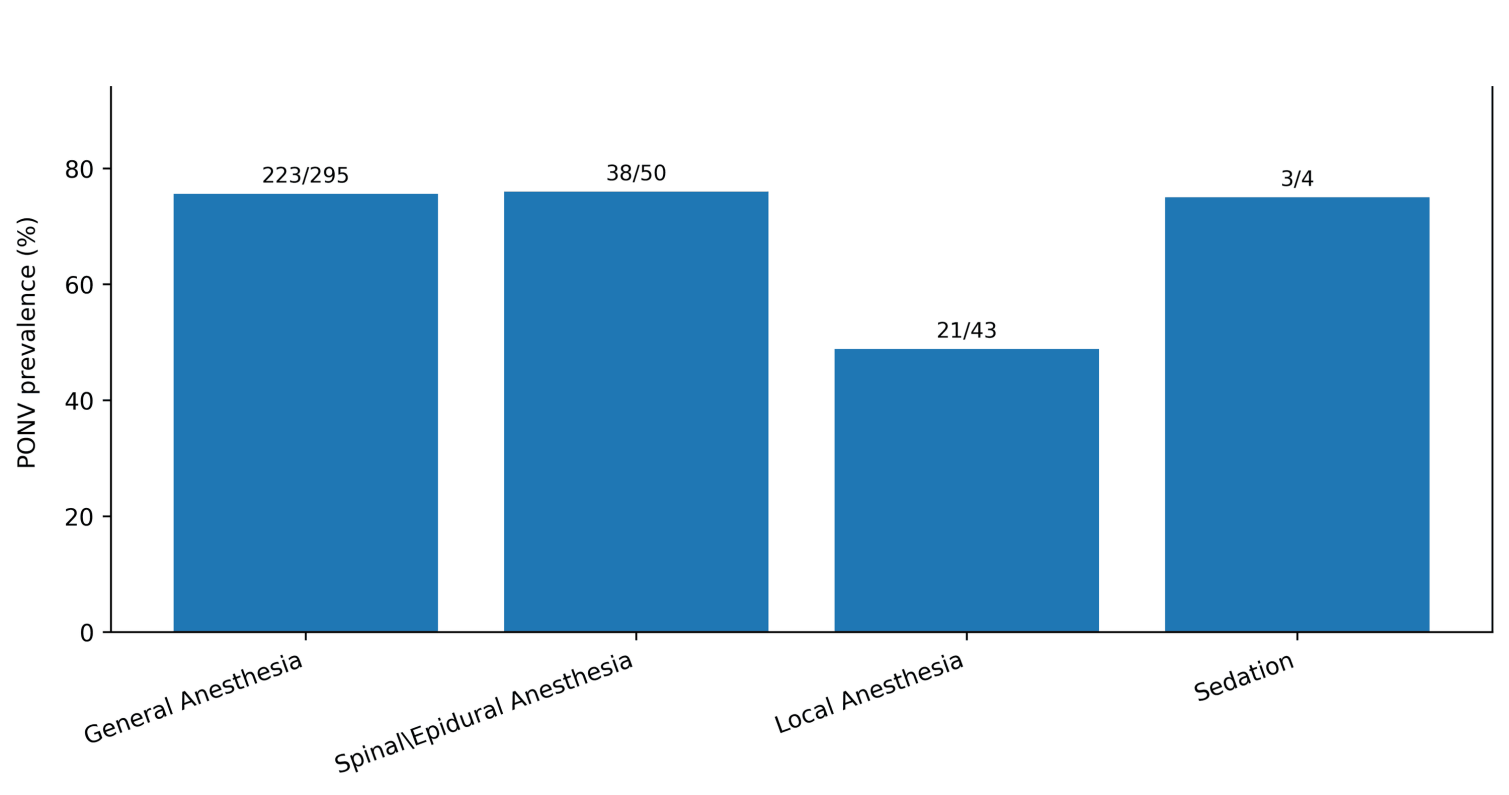
PONV prevalence by anesthesia type Bars show the percentage of participants with PONV (RINVR total >0) by anesthesia type (n=392) [7]. PONV: postoperative nausea and vomiting; RINVR: Rhodes index of nausea, vomiting, and retching

**Figure 5:**
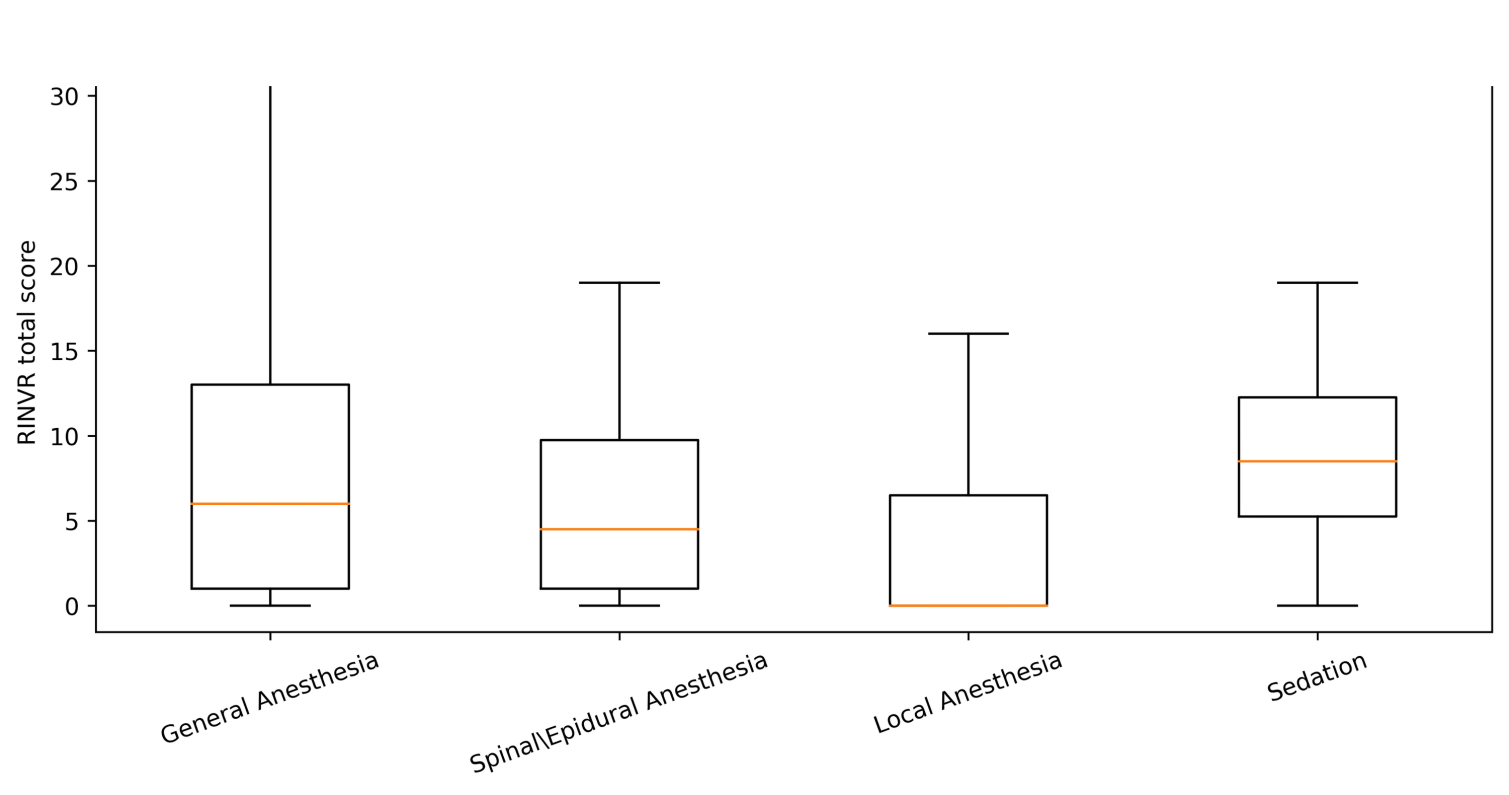
Distribution of total RINVR scores by anesthesia type Total RINVR score by anesthesia type. Box plot of total RINVR scores (0-32) by anesthesia type (n=392). The box represents the IQR, and the center line represents the median [7]. RINVR: Rhodes index of nausea, vomiting, and retching; IQR: interquartile range

Participant characteristics are presented in Table 1. PONV occurred in 285 of 392 participants (72.7%), defined as a total RINVR score greater than 0 [7]. Nausea was reported by 262 participants (66.8%), vomiting by 168 (42.9%), retching by 166 (42.3%), and distress related to symptoms by 233 (59.4%) (Table 2). The total RINVR score ranged from 0 to 32, with a mean of 7.7 (SD 7.7) and a median of 6 ( interquartile range (IQR) 0-11) [7]. Severity categories were: none 107 (27.3%), mild 135 (34.4%), moderate 96 (24.5%), severe 41 (10.5%), and great severity 13 (3.3%) (Figure 1). PONV incidence was higher in females than in males (212/276, 76.8% vs. 73/116, 62.9%). This difference was statistically significant (chi-square=7.25, df=1, p=0.007) (Table 3, Figure 2). Total RINVR scores were higher in females (median 6, IQR 1-13) than in males (median 4, IQR 0-9) (Mann-Whitney U test, Z=3.29, p=0.001) (Table 3, Figure 3) [7]. PONV incidence differed by anesthesia type (chi-square=13.87, df=3, p=0.003) (Figure 4). Local anesthesia had the lowest incidence (21/43, 48.8%) compared with general anesthesia (223/295, 75.6%), spinal or epidural anesthesia (38/50, 76.0%), and sedation (3/4, 75.0%) (Table 3). Total RINVR scores differed by anesthesia type (Kruskal-Wallis H=13.93, df=3, p=0.003) (Table 3, Figure 5) [7]. Age group and weight group were not associated with PONV incidence (age: chi-square=4.17, df=6, p=0.654; weight: chi-square=4.73, df=4, p=0.316). Total RINVR scores did not differ by age group or weight group (age: Kruskal-Wallis H=6.93, df=6, p=0.327; weight: Kruskal-Wallis H=6.68, df=4, p=0.154) (Table 3) [7]. Age was not correlated with total RINVR score (Spearman’s rho=-0.113, p=0.654) [7].

**Table 1:** Participant characteristics (n=392) Overall, 285 out of 392 patients experienced some degree of postoperative nausea and/or vomiting, corresponding to an overall PONV prevalence of 72.7%. PONV: postoperative nausea and vomiting

Variable	Category	n (%)
Gender	Male	116 (29.6%)
	Female	276 (70.4%)
Age group (years)	<18	20 (5.1%)
	18-24	87 (22.2%)
	25-34	79 (20.2%)
	35-44	92 (23.5%)
	45-54	66 (16.8%)
	55-64	40 (10.2%)
	65+	8 (2.0%)
Weight group (kg)	<50	33 (8.4%)
	50-69	125 (31.9%)
	70-79	99 (25.3%)
	80-89	68 (17.3%)
	90+	67 (17.1%)
Anesthesia type	General anesthesia	295 (75.3%)
	Spinal or epidural	50 (12.8%)
	Local anesthesia	43 (11.0%)
	Sedation	4 (1.0%)
Type of surgery	General surgery	129 (32.9%)
	Obstetrics or gynecological surgery	103 (26.3%)
	ENT surgery	56 (14.3%)
	Orthopedic surgery	42 (10.7%)
	Urological surgery	23 (5.9%)
	Plastic surgery	18 (4.6%)
	Ophthalmic surgery	10 (2.6%)
	Cardiothoracic surgery	7 (1.8%)
	Neurosurgery	2 (0.5%)
	Missing or not reported	2 (0.5%)

**Table 2:** PONV outcomes and RINVR severity categories The table summarizes PONV outcomes and symptom severity among the 392 participants. PONV was defined as any total RINVR score greater than 0. Severity categories were based on total RINVR score ranges: 0 (no symptoms), 1-8 (mild), 9-16 (moderate), 17-24 (severe), and 25-32 (great/severe) [7]. PONV: postoperative nausea and vomiting; RINVR: Rhodes index of nausea, vomiting, and retching

Outcome	n	%
Any PONV (RINVR total >0)	285	72.70%
Any nausea	262	66.80%
Any vomiting	168	42.90%
Any retching	166	42.30%
Any distress (due to PONV)	233	59.40%
RINVR severity: 0 (no symptoms)	107	27.30%
RINVR severity: 1-8 (mild)	135	34.40%
RINVR severity: 9-16 (moderate)	96	24.50%
RINVR severity: 17-24 (severe)	41	10.50%
RINVR severity: 25-32 (great/severe)	13	3.30%

**Table 3:** Bivariate associations between patient factors and PONV incidence and severity Bivariate associations between patient factors and PONV incidence and severity (n=392). Incidence p-values and chi-square statistics were obtained using chi-square tests (Yates’ correction applied for 2 × 2 tables). RINVR p-values were derived from the Mann-Whitney U test (gender) or the Kruskal-Wallis test (anesthesia type, age group, and weight group) [7]. PONV: postoperative nausea and vomiting; RINVR: Rhodes index of nausea, vomiting, and retching; IQR: interquartile range

Variable	Category	PONV positive n/N (%)	Incidence test statistic	Incidence p-value	RINVR median (IQR)	Severity test statistic	Severity p-value
Gender	Male	73/116 (62.9%)	χ²=7.25, df=1	0.007	4 (0-9)	Z=3.29	0.001
Gender	Female	212/276 (76.8%)			6 (1-13)		
Anesthesia type	General anesthesia	223/295 (75.6%)	χ²=13.87, df=3	0.003	6 (1-13)	H=13.93, df=3	0.003
Anesthesia type	Spinal/epidural	38/50 (76.0%)			4 (1-10)		
Anesthesia type	Local anesthesia	21/43 (48.8%)			0 (0-6)		
Anesthesia type	Sedation	3/4 (75.0%)			8 (5-12)		
Age group	<18	15/20 (75.0%)	χ²=4.17, df=6	0.654	5 (2-11)	H=6.93, df=6	0.327
Age group	18-24	64/87 (73.6%)			8 (0-16)		
Age group	25-34	62/79 (78.5%)			6 (2-11)		
Age group	35-44	67/92 (72.8%)			6 (0-12)		
Age group	45-54	42/66 (63.6%)			5 (0-9)		
Age group	55-64	29/40 (72.5%)			6 (0-11)		
Age group	65+	6/8 (75.0%)			4 (2-5)		
Weight group	<50 kg	20/33 (60.6%)	χ²=4.73, df=4	0.316	6 (0-15)	H=6.68, df=4	0.154
Weight group	50-69 kg	98/125 (78.4%)			7 (1-14)		
Weight group	70-79 kg	70/99 (70.7%)			5 (0-10)		
Weight group	80-89 kg	49/68 (72.1%)			6 (0-11)		
Weight group	≥90 kg	48/67 (71.6%)			6 (0-12)		

## Discussion

This cross-sectional survey indicates a high burden of PONV among postoperative patients in the Qassim region. The overall rate is higher than that commonly reported in general surgical populations, which is often around 20% to 30% [8]. At the same time, rates in higher-risk settings have been reported to approach 70% to 80% [8]. A multicenter bariatric surgery cohort reported early PONV at similarly high levels [9]. Taken together, these comparisons suggest that many patients in our setting fall into higher-risk profiles and would benefit from consistent prevention strategies.

The high burden observed in this study likely reflects a combination of patient- and perioperative-related risk factors. A large proportion of participants received general anesthesia, and the sample included many women, both of which are recognized risk factors in established models [10]. This finding has practical implications. PONV prevention in routine care should begin with structured risk assessment and standardized prophylaxis rather than relying solely on clinician preference.

Female sex was associated with higher PONV incidence and greater symptom burden in this cohort. This direction of association aligns with extensive literature identifying female sex as one of the strongest predictors of PONV [10]. Large-scale analyses report higher odds of PONV in women compared with men, and this effect persists across age groups [10]. Proposed mechanisms include hormonal influences and biological differences in nausea and vomiting pathways. From a clinical perspective, these findings support classifying female patients as higher risk and applying prophylaxis accordingly, especially when other risk factors are present.

Anesthesia type was also associated with both PONV incidence and symptom severity. Evidence consistently identifies volatile inhalational anesthetics as a major modifiable contributor to PONV [10]. In prior syntheses, volatile anesthesia increased the risk of PONV compared with total intravenous anesthesia [10]. General anesthesia may also increase exposure to perioperative opioids and other emetogenic factors. In contrast, regional techniques and propofol-based approaches are linked to lower PONV rates in many settings [10]. These findings support selecting less emetogenic techniques when clinically appropriate and ensuring multimodal prophylaxis when volatile-based general anesthesia is required.

In our analyses, age was not associated with PONV incidence or severity. Some models suggest that PONV risk decreases with increasing age [10]. Other studies, especially in single-institution cohorts or specific procedures, report no independent age effect after adjustment for other factors [11]. Differences in case mix, prophylaxis patterns, and the age distribution within a sample can influence whether an age effect is detectable. In addition, prophylaxis practices may attenuate observed differences between age groups if clinicians target younger patients for more aggressive prevention.

We also did not observe evidence that body weight was associated with PONV incidence or severity. Although earlier hypotheses proposed a higher risk with obesity, more recent evidence does not support a strong independent association. A systematic review found no meaningful differences in PONV across BMI categories and concluded that obesity is not an independent risk factor [12]. These findings support focusing perioperative prevention efforts on stronger predictors, such as sex and anesthetic exposures, rather than weight alone [12].

Beyond incidence, we assessed symptom burden using the RINVR. Severity patterns were consistent with the risk patterns observed for PONV incidence, supporting the internal consistency of findings across outcome definitions. The RINVR has established validity and reliability in postoperative settings [13]. Prior evaluations report strong internal consistency and correlation with emetic episodes [13]. In this study, the use of the RINVR provided details beyond a binary definition of PONV and allowed a more granular assessment of symptom burden. As with any self-reported instrument, responses may be influenced by recall and comprehension, particularly when symptoms are severe. Even so, the instrument remains a practical tool for measuring PONV severity in clinical research [13].

Several limitations should be considered. The cross-sectional design does not permit causal inference. The online, self-reported survey approach introduces potential recall and selection bias. We did not capture several established predictors, including smoking status, prior PONV or motion sickness, perioperative opioid exposure, nitrous oxide use, and procedure duration. The sample was drawn from a single region, which may limit generalizability. These limitations underscore the need for future prospective studies with standardized perioperative documentation and prophylaxis protocols.

Despite these limitations, the study provides baseline evidence on PONV burden in a Middle Eastern surgical population and identifies clear targets for improvement. Future work should follow patients prospectively through the first 24 to 48 hours postoperatively, record anesthetic and analgesic exposures in detail, and measure adherence to consensus prophylaxis guidelines. Interventional studies comparing total intravenous anesthesia with inhalational techniques, or testing combination antiemetic regimens in higher-risk groups, would directly inform local practice. This progression from prevalence measurement to targeted prevention studies is needed to improve postoperative comfort and recovery.

## Conclusions

PONV was common among postoperative patients in the Qassim region, affecting 72.7% of participants. Female sex and anesthesia type were associated with higher PONV incidence and higher RINVR scores, whereas age and weight were not associated with PONV in this sample. These findings support routine PONV risk assessment and targeted prophylaxis for higher-risk patients, including multimodal antiemetic strategies and the use of less emetogenic anesthetic techniques when feasible. Future prospective studies should evaluate local prophylaxis practices and interventions aimed at reducing the burden of PONV.
